# AstraZeneca COVID-19 vaccine induces robust broadly cross-reactive antibody responses in Malawian adults previously infected with SARS-CoV-2

**DOI:** 10.1186/s12916-022-02342-z

**Published:** 2022-03-28

**Authors:** Marah G. Chibwana, Thandeka Moyo-Gwete, Gaurav Kwatra, Jonathan Mandolo, Tandile Hermanaus, Thopisang Motlou, Nonkululeko Mzindle, Frances Ayres, Mphatso Chaponda, Godwin Tembo, Percy Mwenechanya, Ndaona Mitole, Chisomo Jassi, Raphael Kamng’ona, Louise Afran, David Mzinza, Henry C. Mwandumba, Stephen B. Gordon, Khuzwayo Jere, Shabir Madhi, Penny L. Moore, Robert S. Heyderman, Kondwani C. Jambo

**Affiliations:** 1 Malawi-Liverpool-Wellcome Trust Clinical Research programme (MLW), Blantyre, Malawi; 2 National Institute for Communicable Diseases of the National Health Laboratory Services, Johannesburg, South Africa; 3 MRC Antibody Research Unit, School of Pathology, University of the Witwatersrand, Johannesburg, South Africa; 4 Respiratory and Meningeal Pathogens Research Unit, University of the Witwatersrand, Johannesburg, South Africa; 5 Department of Science/ National Research Foundation: Vaccine Preventable Diseases, University of the Witwatersrand, Faculty of Health Science, Johannesburg, South Africa; 6 Department of Clinical Microbiology, Christian Medical College, Vellore, India; 7 Liverpool School of Tropical Medicine, Liverpool, UK; 8 University of Liverpool, Liverpool, UK; 9 NIHR Global Health Research Unit on Mucosal Pathogens, Research Department of Infection, Division of Infection and Immunity, University College London, London, UK

**Keywords:** SARS-CoV-2, Spike, RBD, AstraZeneca COVID-19 vaccine, Antibodies, VOC

## Abstract

**Background:**

Binding and neutralising anti-Spike antibodies play a key role in immune defence against SARS-CoV-2 infection. Since it is known that antibodies wane with time and new immune-evasive variants are emerging, we aimed to assess the dynamics of anti-Spike antibodies in an African adult population with prior SARS-CoV-2 infection and to determine the effect of subsequent COVID-19 vaccination.

**Methods:**

Using a prospective cohort design, we recruited adults with prior laboratory-confirmed mild/moderate COVID-19 in Blantyre, Malawi, and followed them up for 270 days (*n* = 52). A subset of whom subsequently received a single dose of the AstraZeneca COVID-19 vaccine (ChAdOx nCov-19) (*n* = 12). We measured the serum concentrations of anti-Spike and receptor-binding domain (RBD) IgG antibodies using a Luminex-based assay. Anti-RBD antibody cross-reactivity across SARS-CoV-2 variants of concern (VOC) was measured using a haemagglutination test. A pseudovirus neutralisation assay was used to measure neutralisation titres across VOCs. Ordinary or repeated measures one-way ANOVA was used to compare log10 transformed data, with *p* value adjusted for multiple comparison using Šídák's or Holm-Šídák's test.

**Results:**

We show that neutralising antibodies wane within 6 months post mild/moderate SARS-CoV-2 infection (30–60 days vs. 210–270 days; Log ID_50_ 6.8 vs. 5.3, *p* = 0.0093). High levels of binding anti-Spike or anti-RBD antibodies in convalescent serum were associated with potent neutralisation activity against the homologous infecting strain (*p* < 0.0001). A single dose of the AstraZeneca COVID-19 vaccine following mild/moderate SARS-CoV-2 infection induced a 2 to 3-fold increase in anti-Spike and -RBD IgG levels 30 days post-vaccination (both, *p* < 0.0001). The anti-RBD IgG antibodies from these vaccinated individuals were broadly cross-reactive against multiple VOCs and had neutralisation potency against original D614G, beta, and delta variants.

**Conclusions:**

These findings show that the AstraZeneca COVID-19 vaccine is an effective booster for waning cross-variant antibody immunity after initial priming with SARS-CoV-2 infection. The potency of hybrid immunity and its potential to maximise the benefits of COVID-19 vaccines needs to be taken into consideration when formulating vaccination policies in sub-Saharan Africa, where there is still limited access to vaccine doses.

## Background

The emergence of SARS-CoV-2 (severe acute respiratory syndrome coronavirus 2) variants of concern (VOCs) has contributed to the increased morbidity and mortality caused by the COVID-19 (coronavirus disease 2019) pandemic [1]. Among the growing number of VOCs, the beta (B.1.351) variant was first detected in South Africa and drove the second SARS-CoV-2 epidemic wave in southern Africa, which was associated with a substantial increase in cases and deaths compared to the first wave [2]. The beta variant bears genetic changes in the functional domain of the SARS-CoV-2 spike (S) protein including substitutions in the receptor-binding domain (RBD) (E484K, N501Y and K417N), four substitutions and a deletion in N-terminal domain (NTD) (L18F, D80A, D215G, L242H and R246I) and substitutions in S2 (D614G and A701V) regions [2]. Consequentially, variations in the RBD of the Spike protein, a classic feature of VOCs, not only increases affinity for the ACE2 human receptor [3, 4] but also aids immune evasion [5].

Neutralisation activity against the beta variant was reduced by 13-fold in convalescent sera from individuals infected with the original D614G variant [6, 7]. Furthermore, vaccine-induced antibody neutralisation by the AstraZeneca COVID-19 vaccine was reduced against the beta variant compared to the original D614G variant [8]. As a result, AstraZeneca COVID-19 vaccine showed poor efficacy against mild/moderate beta variant infection [8], but efficacy against severe COVID-19 was not assessed because of the small number of outcomes, while, in a real-life setting, vaccine effectiveness against any symptomatic disease 21 days post first dose of the AstraZeneca COVID-19 vaccine was 50% against the beta/gamma (P.1) variants and 70% and 72% against the delta (B.1.617.2) and alpha (B.1.1.7) variants, respectively [9]. However, vaccine effectiveness against hospitalisation or death was 82% against the beta/gamma variants, and 87% and 90% against the delta and alpha variants, respectively [9]. These studies highlight the immune escape potency of the beta variant, which has now also been seen with other VOCs that have appeared since, including the delta and omicron (B.1.1.529) variants.

Multiple studies have reported that COVID-19 vaccination of individuals who were previously infected with SARS-CoV-2 induces robust cellular and antibody responses [9–11], termed hybrid immunity [12]. Receptor-binding domain (RBD)-specific memory B cells were increased 5- to 10-fold in hybrid immunity compared with natural infection or vaccination alone in two studies [10, 11]. Neutralising antibody titres induced following a single COVID-19 mRNA vaccine dose in previously infected individuals was 20-fold higher than after two doses of same vaccine in naïve individual. Moreover, these antibodies were shown to be cross-neutralising across multiple VOCs, including alpha and beta [10, 13–15]. Recently, it has been shown that breakthrough infections in vaccinated individuals also demonstrate a hybrid immunity enhanced phenotype [16, 17], observed in individuals vaccinated following prior SARS-CoV-2 infection [10, 11, 18]. The increase in cross variant-neutralising antibodies induced by hybrid immunity is thought to be due to a recall response of diverse and high-quality memory B cells generated against natural infection [12]. However, most of these studies have focused on mRNA vaccines, relied on primary infection induced by the ancestral variant, and have been done in non-African populations. To our knowledge, the only published adenovirus-based vaccine study done in an African population was done using the Johnson and Johnson Ad26.COV2.S single-dose vaccine in South Africa, which showed vaccination following prior infection significantly boosts spike-binding antibodies, antibody-dependent cellular cytotoxicity, and neutralising antibodies against D614G, beta, and delta VOCs [18].

Here, we report an assessment of the dynamics of anti-SARS-CoV-2 antibodies in an African adult population with prior SARS-CoV-2 infection and subsequently receiving a single dose of AstraZeneca COVID-19 vaccine. These data offer important insights on natural and vaccine-induced antibody responses against VOCs and highlight the potency of hybrid immunity induced after vaccination with an adenovirus-based vaccine in previously mild/moderate SARS-CoV-2 infected adults.

## Methods

### Study setting and population

Using a prospective study design, recovered mild/moderate COVID-19 adult patients were recruited during the first two epidemic waves in Malawi (Blantyre City, southern region) and followed up every 30 days for a maximum of 270 days. We used a convenience sampling approach, whereby the study was advertised electronically and by word of mouth, with support from the Blantyre District Health Office and Malawi-Liverpool-Wellcome programme. Inclusion criteria for the study included being a Blantyre resident, aged between 18 and 65 years old, and having previous history of laboratory-confirmed diagnosis of COVID-19 not less than 28 days at the time of recruitment. The exclusion criteria included withholding consent and having symptoms suggestive of COVID-19 at time of recruitment. Peripheral blood samples were collected at recruitment and subsequent follow-ups. We used electronic case report forms (eCRFs) to collect clinical history and demographic data. The first wave in Malawi peaked in July 2020 and the second wave peaked in January 2021, driven by the original variant and Beta variant, respectively [19, 20].

### Semi-quantitative SARS-CoV-2 Spike (S) and receptor-binding domain (RBD) IgG antibody enzyme-linked immunosorbent assay

The SARS-CoV-2 original (D614G) spike and RBD proteins were expressed in human embryonic kidney (HEK) 293F suspension cells by transfecting the cells with the spike plasmid. After incubating for 6 days at 37 °C, 70% humidity and 10% CO_2_, proteins were purified using a nickel resin followed by size-exclusion chromatography. Relevant fractions were collected and flash-frozen until use. Spike or RBD protein (2 μg/ml) was used to coat 96-well, high-binding plates and incubated overnight at 4 °C. The plates were incubated in a blocking buffer consisting of 5% skimmed milk powder, 0.05% Tween 20, 1x PBS. Plasma samples were diluted to a 1:100 starting dilution in blocking buffer and added to the plates. Secondary antibody was diluted to 1:3000 in blocking buffer and added to the plates followed by TMB substrate (Thermofisher Scientific). Upon stopping the reaction with 1 M H_2_SO_4_, absorbance was measured at a 450-nm wavelength. In all instances, the CR3022 mAb was used as a positive control and palivizumab was used as a negative control.

### Luminex-based quantitation of SARS-CoV-2 full-length spike and RBD IgG antibodies

The assay was performed as previously reported [21]. In brief, the expression plasmid encoding for SARS-CoV-2 RBD and full-length Spike were obtained from the Florian Krammer, Mount Sinai, USA. The recombinant trimeric Spike and RBD proteins were expressed as described previously [22] and were coupled to the magnetic microsphere beads (Bio-Rad, USA) using a two-step carbodiimide reaction [23]. An in-house references serum was developed by pooling convalescent serum from adult COVID-19 positive patients. This interim reference serum was calibrated against research reagent NIBSC 20/130 distributed by the National Institute for Standards and Biological Control (NIBSC) for the purpose of development and evaluation of serological assays for the detection of antibodies against SARS-CoV-2 (NIBSC, Potters Bar, UK). The binding antibody units (BAU) values assigned to in-house reference serum were 1242 BAU/mL and 2819 BAU/mL for RBD and full-length Spike IgG, respectively. Serum samples collected before 2020 were used for the analysis of assay specificity. Values of 26 BAU/mL and 32 BAU/mL were selected as the threshold indicative of SARS-CoV-2 antibodies, based on the highest value of RBD and full-length Spike IgG in samples from pre-COVID-19. Sensitivity of the assay in detecting past or current infection was assessed using serum samples obtained from randomly selected participants (*n* = 15) who tested SARS-CoV-2 PCR positive and who had serial sampling before and after post-symptom onset, including cases with mild-moderate illness and asymptomatic infections. The sensitivity of the IgG assay was 75% for samples taken 7–14 days and 100 % for samples taken above 14 days following the PCR positive for SARS-CoV-2 for both RBD and full-length Spike IgG. The assay was also evaluated against a COVID-19 convalescent plasma panel (NIBSC code 20/118) intended for the development and evaluation of serological assays for the detection of antibodies against SARS-CoV-2. The in-house characterisation of the plasma panel was in line with recommended criteria with 20/120 having the highest anti-RBD antibody titre followed by 20/122, which had a mid-antibody titre, 20/124 and 20/126 had the lowest titres, and 20/128 as negative. The optimal serum/plasma and secondary antibody dilutions for the assay were 1:100 and 1:200, respectively. The over-range samples were re-tested at higher dilutions (1:200-1:1000). Samples were analysed in duplicate, and each plate included two in-house control sera. Bead fluorescence was read with the Bio-Plex 200 instrument (Bio-Rad) using Bio-Plex manager 5.0 software (Bio-Rad).

### Haemagglutination test for detection of antibodies to SARS-CoV-2 variants of concern

The haemagglutination test (HAT) has been developed in Prof Alain Townsend’s (AT) laboratory at the University of Oxford and uses the IH4-RBD. The IH4-RBD reagent is based on the camelid nanobody VHH-IH4, linked to the RBD of SARS-2 Spike protein. IH4 is specific for a conserved epitope on Glycophorin A, comprised of residues 52–55 (YPPE). The IH4-RBD fusion was designed by AT and has been produced by Absolute Antibody (Oxford, UK) in bulk (1g). One milligram is enough for 10,000 tests (100 ng/well). The RBD proteins were derived from the original D614G strain and the four variants of concern, namely alpha, beta, gamma, and delta. We conducted the assay as previously reported [24, 25]. In brief, O negative red blood cells obtained from the Malawi Blood Transfusion Services were diluted in phosphate-buffered saline (PBS) at 1:20 and plated in V-bottomed 96 well plates. Doubling dilution of serum samples starting from 1:20 were added to the plate until 1:640 dilution. CR3022 (a human monoclonal antibody isolated from a SARS recovered patient) and EY-6A (an antibody isolated from a COVID-19 recovered patient) reagents were used as positive controls, as they all bind to similar epitopes on RBD of all the variants, allowing cross-linking between red blood cells (RBCs) labelled with IH4-RBD. IH4-RBD was added to each well and RBCs were allowed to settle for an hour. The plate was then tilted for at least 30 seconds and photographed. The HAT titre was defined by the last well in which the teardrop fails to form. Partial teardrop was regarded as negative.

### Pseudovirus neutralisation assay

Samples that were seropositive for anti-Spike binding antibodies in the semi-quantitative ELISA were screened for neutralising activity as previously described [6, 18]. SARS-CoV-2-pseudotyped lentiviruses were prepared by co-transfecting the HEK 293T cell line with either the SARS-CoV-2 original spike (D614G) or the SARS-CoV-2 beta or delta spike plasmids in conjunction with a firefly luciferase encoding pNL4 lentivirus backbone plasmid. The parental plasmids were kindly provided by Drs. Elise Landais and Devin Sok (The International AIDS Vaccine Initiative (IAVI), USA). For the neutralisation assay, heat-inactivated seropositive serum samples were incubated with the SARS- CoV-2 pseudotyped virus for 1 h at 37 °C, 5% CO_2_. Subsequently, 1 × 104 HEK 293 T cells that were engineered to over-express ACE-2, kindly provided by Dr. Michael Farzan (Scripps Research), were added and incubated at 37 °C, 5% CO_2_ for 72 h upon which the luminescence of the luciferase gene was measured.

### Statistical analysis

Data visualisation and statistical analyses were performed in GraphPad Prism software (version 9.1.2). The antibody binding and neutralisation activity data were log10 transformed. Ordinary or repeated measures one-way ANOVA was used to compare log10 transformed data, with *p* value adjusted for multiple comparisons using Šídák’s or Holm-Šídák’s multiple comparisons test. Effects were considered statistically significant when the *p*-value was less than 0.05.

## Results

### Participant demographic and clinical characteristics

Here, we studied a cohort of individuals that had recovered from mild/moderate COVID-19 (*n* = 52) (Table 1). The median time from laboratory-confirmed COVID-19 diagnosis to recruitment was 70 days (59–87) for the first wave and 63 days (39–71) for the second wave. Based on genomic surveillance data from Malawi [19], it is expected that the participants were infected by either ancestral variants in the first wave or the beta variant in the second wave. However, as genomic data were not collected as part of this study, we cannot completely rule out the possibility of infections from other variants of concern (VOCs) during the second wave, especially the alpha variant. Eight out of 52 individuals reported having a comorbidity, defined as diabetes, hypertension, or HIV. Twelve (23% [12/52]) of the study participants were vaccinated with one dose of the AstraZeneca COVID-19 vaccine during the follow-up period of the study as part of the routine vaccination programme. COVID-19 vaccination using the AstraZeneca COVID-19 vaccine was rolled out in Malawi from 11 March 2021 with 768,212 fully vaccinated as of 24 January 2022.

**Table 1:** Participant demographic and clinical characteristics

Sociodemographic and clinical characteristics	Mild/moderate COVID-19 ***n*** = 52
**Sex**	
Male, *n* (%)	30 (58%)
Female, *n* (%)	22 (42%)
**Age in years**	
Median (range)	36 (22–42)
**Days from laboratory-confirmed SARS-CoV-2 diagnosis to enrolment**	
First wave, *n*; median (IQR)	30; 70 (59–87)
Second wave, *n*; median (IQR)	22; 63 (39–71)
**COVID-19 severity**	
^a^Mild	28 (54%)
^b^Moderate	19 (37%)
Missing data	5 (9%)
**Comorbidities (diabetes, hypertension or HIV)**	
Yes	8 (15%)
No	44 (85%)

^a^Positive SARS-CoV-2 PCR without evidence of viral pneumonia or hypoxia

^b^Positive SARS-CoV-2 PCR with clinical signs of pneumonia (fever, cough, dyspnea, fast breathing) but no signs of severe pneumonia, including SpO_2_ > 89% on room air

### SARS-CoV-2 pseudovirus neutralisation activity wanes over time

**Fig. 1:**
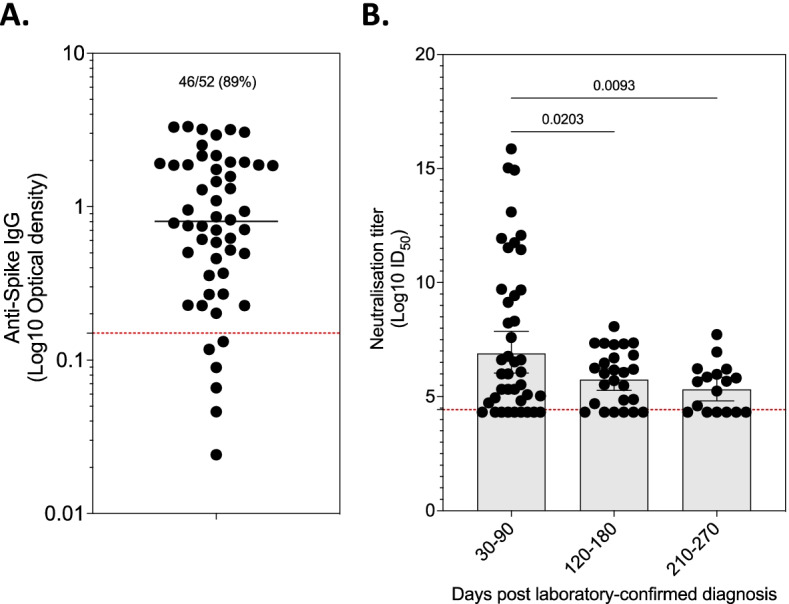
Antibody responses following mild/moderate SARS-CoV-2 infection. **A** Presence of anti-Spike IgG in convalescence serum (*n* = 52). **B** Pseudovirus neutralisation activity in anti-Spike IgG seropositive sera following mild/moderate COVID-19 (*n* = 45). Data was log 10 transformed and statistics were calculated using ordinary one-way ANOVA and *p* value adjusted for multiple comparison using Šídák’s multiple comparisons test. Vertical bars represent geometric mean and horizontal bars represent 95% confidence intervals. A *p* < 0.05 was regarded as statistically significant

We tested the longevity of the neutralising activity in convalescent serum using a pseudovirus neutralising assay against the original D614G variant and the beta variant. Only 46 out of 52 individuals met the criteria for pseudovirus neutralisation assays (Fig. 1A), but one sample was not tested. Anti-Spike seropositive sera from individuals infected during the first wave were assessed for neutralisation activity against the D614G variant, while that from individuals infected in the second wave was tested against the beta variant. The neutralisation activity at 30–90 days was higher than that at 120 to 180 days (*p* = 0.0203) or 210 to 270 days (*p* = 0.0093), but there was no statistically significant difference between 120 to 180 days and 210 to 270 days (Fig. 1B). These data show significant decline in serum pseudovirus neutralisation activity within 6 months post laboratory-confirmed mild/moderate COVID-19.

### Low pseudovirus neutralisation activity in convalescent serum is associated with SARS-CoV-2 reinfection

**Fig. 2:**
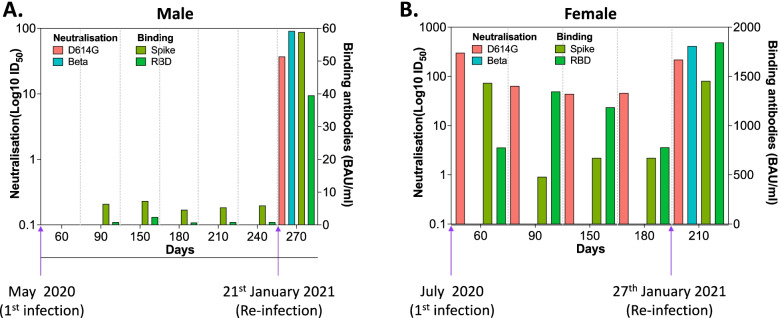
SARS-CoV-2 specific antibody responses in re-infected individuals. Two participants had a laboratory-confirmed SARS-CoV-2 reinfection during the follow-up period of the study. They were both re-infected during the beta variant wave. Kinetics of anti-Spike, anti-RBD, and neutralisation activity in serum of **A** 46-year-old male and **B** 49-year-old female. S, full-length Spike; RBD, receptor-binding domain; SARS-CoV-2, severe acute respiratory syndrome coronavirus 2

We observed two laboratory-confirmed SARS-CoV-2 reinfections during the study period. The first re-infected participant was a 46-year-old male who had mild COVID-19 during the first wave and was recruited into the study at 90 days post diagnosis (Fig. 2A). His last visit before PCR-confirmed reinfection was day 240, at which he had undetectable pseudovirus neutralisation activity (D614G, ID_50_ < 20, beta, ID_50_ < 20) and anti-Spike and anti-RBD IgG antibody levels of 5.9 BAU/ml and 0.8 BAU/ml, respectively. At his scheduled monthly visit (day 270), a noticeable increase in anti-Spike (58.8 BAU/ml) and anti-RBD (39.5 BAU/ml) was observed confirming reinfection. His reinfection episode was mild as the primary infection. Consistent with the time of reinfection (second wave), he had higher pseudovirus neutralisation titres against the beta variant than D614G (ID_50_ 91 vs 37). The second re-infected participant was a 49-year-old female who had moderate COVID-19 during the first wave and was recruited at 60 days post diagnosis (Fig. 2B). Her last visit before reinfection was day 180, at which she had low pseudovirus neutralisation activity (D614G, ID_50_ 46, beta, ID_50_ < 20) and anti-Spike and anti-RBD IgG antibody levels of 669.8 BAU/ml and 778.7 BAU/ml, respectively. At her scheduled monthly visit of day 210, a noticeable increase in anti-Spike (1453.4 BAU/ml) and anti-RBD (1844.5 BAU/ml) was also observed confirming reinfection. Her reinfection episode was moderate like the primary infection. Again, consistent with a second wave infection, she had higher pseudovirus neutralisation titres against the beta variant than the D614G variant (ID_50_ 414 vs 219). These data demonstrate that loss or absence of variant-specific pseudovirus neutralising antibodies was associated with reinfection in these two participants.

### High levels of anti-Spike and RBD IgG antibodies are associated with potent pseudovirus neutralising activity against infecting variant

**Fig. 3:**
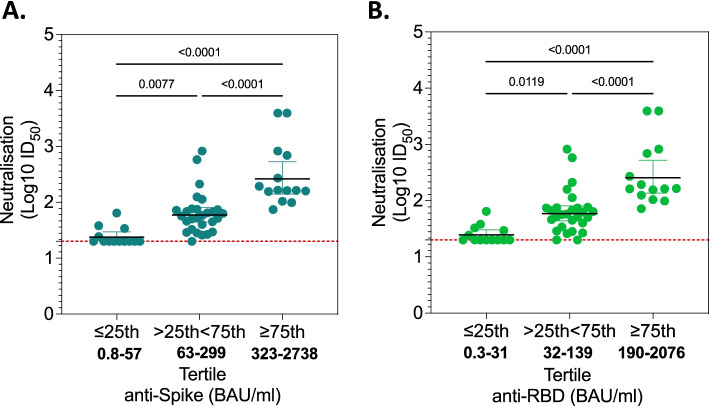
Neutralisation activity against the original variant in recovered mild/moderate COVID-19 patients with various levels of anti-Spike and RBD IgG antibodies. Magnitude of neutralisation activity against the original variant (D614G) in first wave sera with varying concentrations of **A** anti-Spike IgG and **B** anti-RBD IgG antibodies. The Spike and RBD protein antigens were from the original D614G variant. Vertical bars represent geometric mean and horizontal bars represent 95% confidence intervals. Data was log 10 transformed and statistics were calculated using ordinary one-way ANOVA and *p* value adjusted for multiple comparison using Šídák’s multiple comparisons test (*n* = 30). A *p* < 0.05 was regarded as statistically significant. S, full-length Spike; RBD, receptor-binding domain; SARS-CoV-2, severe acute respiratory syndrome coronavirus 2

To obtain further insights on the association between binding antibody levels and neutralisation activity, we quantified the concentrations of anti-Spike and anti-RBD IgG antibodies in the convalescent sera from the first wave using a single-plex bead-based immunoassay [21]. We grouped the data into three tertiles based on the antibody concentrations, low (≤ 25th centile), medium (> 25th < 75th centile) and high (≥ 75th centile), and then assessed the level of pseudovirus neutralisation activity against D614G in these three groups. The level of pseudovirus neutralisation activity was higher in those with concentrations of anti-Spike or anti-RBD antibodies in the medium centile (anti-Spike, *p* = 0.0077; anti-RBD, *p* = 0.0119) and high centile (anti-Spike, *p* < 0.0001; anti-RBD, *p* < 0.0001), compared to those in low centile group (Fig. 3A, B). The level of pseudovirus neutralisation activity was higher in those with levels of anti-Spike or anti-RBD antibodies in the high centile compared to medium centile (anti-Spike, *p* < 0.0001; anti-RBD, *p* < 0.0001) (Fig. 3A, B). These data show that high concentrations of anti-Spike and anti-RBD binding antibodies in convalescent serum from mild/moderate COVID-19 was indicative of potent pseudovirus neutralisation activity against the infecting variant.

### Partial vaccination of recovered mild/moderate COVID-19 patients induces robust antibody responses

**Fig. 4:**
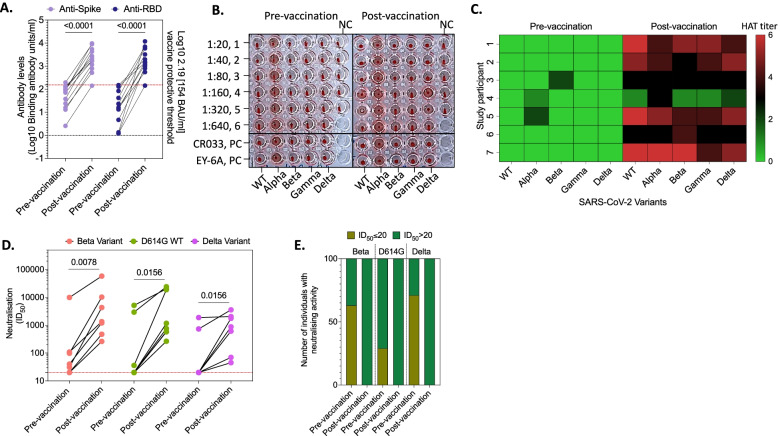
Antibody responses following adenovirus COVID-19 vaccination in previously infected adults. **A** Concentration of anti-Spike and anti-RBD IgG antibodies pre- and post-vaccination with a single dose of AstraZeneca COVID-19 vaccine. All antigens were from the original strain. **B** Representative plate of the haemagglutination test against the wild type, alpha, beta, gamma, and delta variants using pre- and post-vaccination sera. The sera were diluted 2-fold for 6 dilutions. CR033 and EY-6A are used are positive control, and red blood cells from blood group O negative are used as negative control. **C** Heat map showing collated data of HAT titres against the wild type, alpha, beta, gamma, and delta variants in pre- and post-vaccination sera. **D** Magnitude of neutralisation activity against the beta, D614G, and delta variants in pre- and post-vaccination sera. **E** Number of individuals with neutralisation activity against Beta or D614G or Delta variants in pre- and post-vaccination sera. Statistics were calculated using RM one-way ANOVA and *p* values adjusted for multiple comparison using Holm-Šídák’s multiple comparisons test. A *p* < 0.05 was regarded as statistically significant. S, full length Spike; RBD, receptor-binding domain; HAT, haemagglutination test; NC, negative control; PC, positive control; SARS-CoV-2, severe acute respiratory syndrome coronavirus

We next assessed whether vaccination with a single dose of the AstraZeneca COVID-19 vaccine in individuals who previously had mild/moderate COVID-19 could impact humoral responses against SARS-CoV-2, as has been observed with other COVID-19 vaccines [9–11, 18]. A total of 12 participants in our study were vaccinated, as part of the routine national COVID-19 vaccine roll-out, at a median of 60 days (IQR 60–75) post laboratory-confirmed diagnosis of mild/moderate COVID-19. All the individuals were recruited in the second wave and hence were likely to have been infected with the beta variant. The median duration from vaccination to post-vaccination sample collection was 25 days (IQR 21–28). Antibody responses post-vaccination were assessed at a single time point. We observed a 2-fold (*p* = 0.0031) and 4-fold (*p* < 0.0001) increase in the levels of anti-Spike and RBD IgG antibodies against the original D614G variant following vaccination, respectively (Fig. 4A). Most of the individuals (92% [11/12]) had antibody levels beyond the suggested protective level of 154 BAU/ml [26] following vaccination (Fig. 3A). In a subset of the vaccinated participants with paired samples (*n* = 7), we assessed whether vaccination enhanced the breadth of the antibody response, defined as the presence of binding antibodies in serum against multiple VOCs. We used a recently published SARS-CoV-2 RBD haemagglutination test (HAT) that correlates well with neutralisation activity [24, 25]. We tested reactivity against D614G, alpha, beta, gamma, and delta variants in pre-vaccination and post-vaccination serum. Reactivity to all VOCs was observed in post-vaccination serum but not in pre-vaccination serum (Fig. 4B, C). These data show improved antibody recognition of RBD variations following vaccination in previously SARS-CoV-2 infected individuals.

To ascertain enhanced neutralisation activity after vaccination, sera from individuals who were infected during the second wave and subsequently vaccinated was tested for pseudovirus neutralisation activity against both the D614G, beta, and delta variants. Pseudovirus neutralisation activity was increased against D614G (*p* = 0.0156), beta (*p* = 0.0078), and delta (*p* = 0.0156) variants (Fig. 4D). Moreover, even in those that had no pre-vaccination pseudovirus neutralisation activity against the D614G (pre-vac 29% vs. post-vac 100%) or beta (pre-vac 63% vs. post-vac 100%) or delta variants (pre-vac 71% vs. post-vac 100%), vaccination rescued pseudovirus neutralisation activity against the variants (Fig. 4E). These data showed that vaccination improved pseudovirus neutralisation activity not only against the infecting variant (beta variant) but also the original D614G and delta variants.

## Discussion

In this study, we report binding and neutralisation antibody responses induced by SARS-CoV-2 infection and augmented by a single dose of the AstraZeneca COVID-19 vaccine. We show that antibody pseudovirus neutralisation activity induced following SARS-CoV-2 infection wanes within 6 months post laboratory-confirmed diagnosis of mild/moderate COVID-19. High concentrations of binding anti-Spike and anti-RBD IgG antibodies are associated with the presence of pseudovirus neutralisation activity in convalescent serum. Most importantly, vaccination with AstraZeneca COVID-19 vaccine in individuals with prior SARS-CoV-2 infection induced robust binding, cross-reactive, and cross-neutralising antibody responses against multiple VOCs.

mRNA vaccination in previously infected individuals induce robust cross-reactive antibody responses against SARS-CoV-2 [9–11]. Consistent with these observations, we show that an adenovirus vaccine elevates levels of anti-Spike and anti-RBD antibodies that are cross-reactive against D614G, alpha, beta, gamma, and delta variants in individuals previously infected with SARS-CoV-2. We also observed cross-neutralisation across VOCs in the vaccinated individuals. In agreement, studies done in the UK (Vaxzervria) and India (COVISHIELD) also show that a single dose of the AstraZeneca COVID-19 vaccine induces high antibody titres in individuals with previous SARS-CoV-2 exposure [13, 27]. Of note, the UK study showed a significant increase in neutralising antibody titres to VOCs including alpha, beta, and gamma, but with the least increase observed for the beta and gamma variants [13]. Considering that in vitro neutralising activity is highly predictive of immune protection from symptomatic SARS-CoV-2 infection [27], presence of cross-neutralising antibodies following vaccination with AstraZeneca COVID-19 vaccine in recovered patients could potentially confer cross-protection against multiple variants.

Anti-Spike IgG antibody concentrations between 60 BAU/ml to 154 BAU/ml following vaccination were recently suggested as a potential threshold for protective immunity against symptomatic COVID-19 [26, 28]. In our study, a single dose of AstraZeneca COVID-19 vaccine resulted in anti-Spike IgG antibodies concentrations of 12 times above the upper limit of the proposed protective threshold of 154 BAU/ml. Moreover, following SARS-CoV-2 infection, we show high pseudovirus neutralisation activity against the infecting variant in individuals with anti-Spike IgG antibodies of greater than 62 BAU/ml. However, levels of pseudovirus neutralising antibodies induced following mild/moderate SARS-CoV-2 infection declined significantly within 6 months post diagnosis, which agrees with published literature [29, 30]. Notably, in those who experienced reinfection in our cohort, the timing of the reinfection occurred at the time point when there was loss of serum pseudovirus neutralisation activity against the reinfecting variant, underscoring the importance of variant-specific pseudovirus neutralising antibodies in protection from SARS-CoV-2 infection. However, in one of the reinfected individuals, the binding anti-Spike and anti-RBD IgG antibody concentrations in the last sample before reinfection were above the threshold of 154 BAU/ml, indicating that binding antibody thresholds may not work universally across variants.

Interestingly, one of the vaccinated individuals did not reach the proposed putative protective threshold of 154 BAU/ml following a single dose of the AstraZeneca COVID-19 vaccine. This individual had the lowest anti-Spike (144 BAU/ml) and anti-RBD (142 BAU/ml) binding antibodies in convalescent serum pre-vaccination among the vaccinated participants and had the lowest breadth in the HAT assay (participant #4). This finding suggests that hybrid immunity may be influenced by the baseline antibody response at the time of vaccination and warrants further investigation.

This study has considerable strengths including the availability of both binding and neutralisation data from an underrepresented sub-Saharan African population; however, there are some important limitations. First, the analysis on longevity was cross sectional, and this could impact the accuracy of our estimates on the duration of binding and neutralisation antibodies. Moreover, we have only measured binding and neutralisation function in this study, but there are other Fc-mediated antibody functions [31], as well as memory T and B cell responses [32, 33], that have previously been implicated to contribute to the control of SARS-CoV-2 infection. Nevertheless, binding and neutralising antibodies are the most well characterised potential correlates of protection (CoP) against COVID-19 to date [26, 28]. Second, the sample size, especially for the vaccinated individuals, is small, and this may limit the generalisability of the findings. However, the consistency of the results across multiple assay platforms and their agreement with other similar published studies in other populations attests to the robustness of the findings and highlights their relevance to the field.

## Conclusions

We report waning of pseudovirus neutralising activity within 6 months post laboratory-confirmed diagnosis of mild/moderate COVID-19 and a robust antibody response following partial vaccination with the AstraZeneca COVID-19 vaccine in adults previously infected with SARS-CoV-2. These data could have implications for COVID-19 vaccination policy as they provide further evidence highlighting the potency of hybrid immunity induced following vaccination with AstraZeneca vaccine, which is the most common vaccine in sub-Saharan Africa. However, there are still outstanding questions that needs to be addressed, which include whether partial vaccination could also induce robust humoral responses in those with prior asymptomatic infection and how long the enhanced hybrid immunity last, as these could help inform subsequent vaccination regimens or timings.

## Data Availability

All data generated or analysed during this study are available upon request.

## Abbreviations

SARS-CoV-2: Severe acute respiratory syndrome coronavirus 2
VOC: Variant of concern
IgG: Immunoglobulin G
RBD: Receptor-binding domain
S: Spike
COVID-19: Coronavirus disease of 2019
BAU: Binding antibody units
NIBSC: National Institute for Standards and Biological Control
PCR: Polymerase chain reactions
IAVI: International AIDS Vaccine Initiative
ACE2: Angiotensin-converting enzyme 2
